# ATR–FTIR spectroscopy reveals involvement of lipids and proteins of intact pea pollen grains to heat stress tolerance

**DOI:** 10.3389/fpls.2014.00747

**Published:** 2014-12-22

**Authors:** Rachid Lahlali, Yunfei Jiang, Saroj Kumar, Chithra Karunakaran, Xia Liu, Ferenc Borondics, Emil Hallin, Rosalind Bueckert

**Affiliations:** ^1^Canadian Light Source, SaskatoonSK, Canada; ^2^Department of Plant Sciences, University of SaskatchewanSaskatoon, SK, Canada

**Keywords:** lipids, protein secondary structures, pollen grains, pea, heat stress, infrared ATR–FTIR spectroscopy

## Abstract

With climate change, pea will be more frequently subjected to heat stress in semi-arid regions like Saskatchewan during flowering. The pollen germination percentage of two pea cultivars was reduced by heat stress (36°C) with an important decrease in cultivar ‘CDC Golden’ compared to ‘CDC Sage.’ Lipids, protein and other pollen coat compositions of whole intact pollen grains of both pea cultivars were investigated using mid infrared (mid-IR) attenuated total reflectance (ATR)–Fourier transform infrared (FTIR) spectroscopy. Curve fitting of ATR absorbance spectra in the protein region enabled estimation and comparison of different protein secondary structures between the two cultivars. CDC Sage had relatively greater amounts of α-helical structures (48.6–43.6%; band at 1654 cm^-1^) and smaller amounts of β-sheets (41.3–46%) than CDC Golden. The CDC Golden had higher amounts of β-sheets (46.3–51.7%) compared to α-helical structures (35.3–36.2%). Further, heat stress resulted in prominent changes in the symmetrical and asymmetrical CH_2_ bands from lipid acyl chain, ester carbonyl band, and carbohydrate region. The intensity of asymmetric and symmetric CH_2_ vibration of heat stressed CDC Golden was reduced considerably in comparison to the control and the decrease was higher compared to CDC Sage. In addition, CDC Golden showed an increase in intensity at the oxidative band of 3015 cm^-1^. These results reveal that the whole pollen grains of both pea cultivars responded differently to heat stress. The tolerance of CDC Sage to heat stress (expressed as pollen germination percentage) may be due to its protein richness with α-helical structures which would protect against the destructive effects of dehydration due to heat stress. The low pollen germination percentage of CDC Golden after heat stress may be also due to its sensitivity to lipid changes due to heat stress.

## INTRODUCTION

Heat stress is one of the key environmental stresses affecting yield of pea cultivars (*Psium sativum* L.) under semi-arid agricultural conditions in Saskatchewan. Heat stress occurs when temperatures are high enough for sufficient time to cause irreversible damage to plant function or development. Heat stress is a complex function of temperature, duration, and rate of increase in temperatures. Hall (1990, 1993) reviewed the detrimental effects of heat stress on the reproductive developments in cowpea, common bean, tomato, wheat, rice, maize, and sorghum. Direct evidence of heat stress effects on grain yield is provided by studies on different plants that are subjected to various increments of elevated temperatures under field conditions in: cowpea (Nielsen and Hall, 1985); Calabrian pine (Tilki and Dirik, 2007); and elm (Cicek and Tilki, 2007).

The sensitivity of a flower to heat stress is influenced by its position on the inflorescence and the presence or absence of “flowers on the stem below it.” In pea, daytime temperatures of 32°C do not immediately result in sterile flowers but result in delayed abortion of reproductive structures in distal flowers, whereas the more proximal flowers in a node are not affected (Guilioni et al., 1997). Moderate heat stress causes changes in the carbon metabolism that is likely to trigger the abortion of reproductive organs in these distal flowers (Guilioni et al., 1997).

Temperatures can also affect pollen grains during transport to and germination upon the stigma, as well as during development in the anther. Mature pollen grains are generally resistant to temperature stress applied after dehiscence, but detailed investigations have been carried out only in a few instances. In tomato, applying mild heat stress after dehiscence reduces fruit set, and the differences in fruit set with controls are not significant (Sato et al., 2002).

A severe heat stress treatment of *Brassica* pollen revealed that pollen grains germinate after exposure to 60°C for 4 h. Seed set after pollination with pollen treated at 75°C or at 60°C for 24 h is reduced, but pollination with other samples led to normal seed set (Rao et al., 1992). One possible explanation for this observation may be that pollen that germinated on the stigma can overcome any effects of the heat treatment. It is also possible that stigmas may be over-pollinated, thereby reducing germination rates. Further, pollination was performed only with heat treated pollen and no selection on pollen for tube growth rate was conducted. Therefore, pollen performance may be affected by mixed pollination as it occurs in natural environments. Accordingly, plants may experience different types of stress at different developmental stages and these mechanisms of response to stress may vary in different tissues and between plant species (Wahid et al., 2007).

Pollen has a wall which is completely different from any other plant structures. The pollen wall is multilayered, derived from both the developing pollen and sporophytic cells of the anther, and consists of material that is highly resistant to degradation, making it an important determinant of pollen viability (Taylor and Hepler, 1997; Wahid et al., 2007). The surface of the pollen wall, also known as the pollen coat in some plant species, consists of materials like tryphine or protein-rich matrix, pollenkitt (Taylor and Hepler, 1997). This sticky substance may contain lipids, proteins and phenolic compounds, and can be present in substantial amounts in entomophilous species. The functions of the pollen coat are to facilitate pollen transfer through sticking to insects and protection against environmental stress, but the lipids and proteins of the pollen coat may also play an important role in the adhesion of pollen grains to the stigma (Dickinson et al., 2000). Studies of mechanisms of tolerance of pea cultivars to heat stress in semi-arid conditions are still in their infancy and the role of the pollen coat in pea tolerance to heat stress is unclear. We hypothesized that heat resistance mechanisms are present in pollen in some pea genotypes. Genotypes with pollen that has heat resistance will maintain pollen vigor and increased pollen germination, allowing for a greater success in fertilization and seed set in warm temperatures. Such a mechanism or mechanisms should be evident and detectable in the physical structure of pollen, by maintained wall integrity, protein and lipid integrity, and ultimately demonstrated in the ability of pollen grains to germinate. Therefore, we proposed for the first time, the use of mid infrared (mid-IR) attenuated total reflectance (ATR)–Fourier transform infrared (FTIR) spectroscopy as a suitable tool to understand pea pollen surface composition and its potential role in tolerance to heat stress.

The FTIR spectroscopy has recently become an established method for quick and reliable analysis of the composition of agricultural materials such as grain, pollen, plants, forages, and soils (Sowa et al., 1991; Chamel and Marechal, 1992; Sowa and Connor, 1995; Ivanova and Singh, 2003; Alonso-Simon et al., 2004; Bernier et al., 2013; Bruckman and Wriessnig, 2013; Zimmermann and Kohler, 2014). The FTIR spectroscopy has also been successfully used for detecting and identifying microorganisms in food products (Oberreuter et al., 2002; Winder and Goodacre, 2004; Filip et al., 2006; Oliveira et al., 2009). The FTIR spectroscopy is advantageous because it has many distinct peaks amenable to spectral interpretation according to diagnostic bands (Buta et al., 1997; Alonso-Simon et al., 2004; Kong and Yu, 2007).

The objective of this work was to use ATR–FTIR spectroscopy to analyze intact pollen grains of two pea cultivars cultivated largely in the province of Saskatchewan. The surface compositions of pollen from control and heat stressed plants of two cultivars, CDC Golden and CDC Sage, were studied and compared. The changes in the pollen surface composition of both cultivars were compared to the differences in pollen development observed for both pea cultivars under controlled conditions.

## MATERIALS AND METHODS

### PLANTS

Pea cultivars, CDC Golden and CDC Sage were tested in this study. Plants were grown in a 2 L volume (three plants per pot) using Sunshine Gro mix and slow-release fertilizer in the phytotron facility at the University of Saskatchewan. Plants were thinned to two plants per pot ~2 weeks after seeding. Plants received the first application of half strength Hoagland’s culture solution (Zhao et al., 2005) at 3 weeks after seeding and the second application at the early flowering stage. The nutrient solution with pH 6.05 ± 0.05 was made of 2.5 ml of the following solution into 1 L distilled water: K_2_PO_4_ (34.84 g L^-1^), KNO_3_ (101.11 g L^-1^), Ca(NO_3_)_2_*H_2_O (236.15g L^-1^), MgSO_4_*7H_2_O (98.59 g L^-1^), trace elements (0.57 g L^-1^ H_3_BO_3_, 0.36 g L^-1^ MnCl_2_*4H_2_O, 0.04 g L^-1^ ZnSO_4_*7H_2_O, 0.016 g L^-1^ CuSO_4_*5H_2_O). Each pot received 500 ml of the solution each time. Soil medium water moisture was monitored carefully to avoid drought stress, and watered as necessary with distilled water. Plants were grown in growth chambers at 24/18°C day/night temperatures with a 16 h photoperiod in each 24 h cycle in a phytotron, and then were transferred to a second growth chamber to expose them to high temperatures for 7 days when the flowers at the second fruiting node of main stem were fully open. The heat stress temperature assessed in this study was 36/18°C day/night. The control plants continued to stay in the 24/18°C regime. Mature pollen grains of CDC Golden and CDC Sage at 24 and 36°C were collected from the slightly opened flowers on the fourth day after plants were exposed to high temperature.

### GERMINATION TEST

*In vitro* pollen germination was evaluated using a pollen germination medium. Pollen grains were collected from plants in the control regime with the temperature of 24/18°C day/night. The medium consisted of 15 g sucrose (C_12_H_22_O_11_), 0.03 g calcium nitrate [Ca(NO_3_)_2_ 4H_2_O], and 0.01 g boric acid (H_3_BO_3_) dissolved in 100 mL of deionized water (Shivanna and Rangaswamy, 1992; Salem et al., 2007). To solidify the medium, 0.5 g of agar was added to medium. Fresh pollen was collected from all anthers of slightly opened flowers at 5–6 h after the 16 h photoperiod began, which compared to 9:00–10:00 am in the field during summer. Pollen grains were collected from the anthers from between 10:00 and 11:00 am, was dusted onto the germination medium on microscope slides, and then microscope slides were placed individually in 9 cm diameter petri dishes with moistened filter paper. The lids of petri dishes were sealed with parafilm to maintain high humidity. The slides were placed in each of two growth chambers with two temperature treatments at 24/18 and 36/18°C day/night temperatures. After 10 h of incubation, pollen grains (100 grains per replication, five replications per treatment) were counted for germination rate using direct microscopic observation. Germinated pollen grains were determined when the length of the pollen tube was more than the diameter of pollens grains (Shivanna and Rangaswamy, 1992; Salem et al., 2007). Pollen germination percentage was calculated by dividing the number of germinated pollens by the total number of pollens and expressed as a percentage. Arcsine transformation was used to normalize percentage data distribution and then analyzed using one-way ANOVA procedure of the Statistical Analysis System (SAS Institute, version 9.1, Cary, NC, USA). Means values were compared using Fisher’s LSD test at statistical significance of *P* ≤ 0.05.

### ATR–FTIR SPECTROSCOPY

Mature pollen samples from four replicates were collected by dusting pollen grains from anthers harvested from fresh flowers ~45 min prior to the ATR reflectance measurement. The pollens on CaF_2_ slides. were kept in a petri dish with moistened filter paper to minimize possible dehydration until the measurement time. All FTIR spectroscopy data were collected at the mid infrared beamline (01B1-1) at the Canadian Light Source using the glowbar source (silicon carbide) as the infrared source. The pollen surface composition was determined by ATR method using a germanium crystal (20x objective, 100 μm crystal surface, angle of incidence = 45°) attached to a Bruker Hyperion, 2000 confocal microscope (Bruker Optics, Ettlingen, Germany) with a KBr beam splitter and liquid nitrogen cooled mercury cadmium telluride (MCT) detector. Each IR spectrum was recorded in the mid infrared range of 4000–800 cm^-1^ at a spectral resolution of 2 cm^-1^. The ATR germanium crystal was in contact with the pollen after applying the standard pressure setting. The penetration depth was further increased by maximizing the infrared signal from the sample during which the pollen grain was crushed. Each sample spectrum is an average of 512 scans, and normalized using a background reflectance spectrum (average of 1024 scans) from a fresh gold surface. Spectra from 15 individual pollen grains per combination (Bruker’s OPUS Pro 7.0 at 1 number of ATR reflexions, 45° angle of incidence, and 1.5 mean refraction index of sample) of cultivar and heat stress treatment were collected and an extended ATR correction was applied for each spectrum before data analysis.

To estimate lipids, phosphate, and carbohydrate contents the ATR spectra were normalized using the protein peak (1700–1600 cm^-1^) and for the protein content analysis the ATR spectra were normalized using the lipid-ester peak at 1740 cm^-1^. The estimation of components such as phosphate and carbohydrate were determined by integrating the area under the specific bands. The area integration were determined using the the OPUS integration method C. This method allowed to choose four points, in which the two points were considered left and the right of the interested band, and another two points were far away for the baseline on each side to get a straight baseline.

### PEA PROTEIN SECONDARY STRUCTURES

The pea protein secondary structure analysis was carried out by curve fitting using a program Kinetics, written by Goormaghtigh et al. (2006) using Matlab (version R2008, Mathworks Inc.). First, the second derivative spectra were calculated from the ATR–FTIR spectra after smoothing over two consecutive points. The absorption bands at low wavenumber were free of features from water vapor as judged from the peaks above 1750 cm^-1^. A straight baseline passing through the ordinate at 1700 and 1610 cm^-1^ was subtracted before the curve fitting. The baseline was again modified by the least-squares curve fitting program which allows for a horizontal baseline to be adjusted as an additional parameter to obtain the best fit. The second derivative spectrum was used to determine the initial peak positions for curve fitting and the peaks were fitted using Voigt functions (Goormaghtigh et al., 1990). In curve fitting, each component is a mixture of Lorentzian and Gaussian lineshapes. The area under the entire band was considered 100% and each component after fitting was expressed as a percent fraction.

## RESULTS

### PEA POLLEN GERMINATION

**FIGURE 1:**
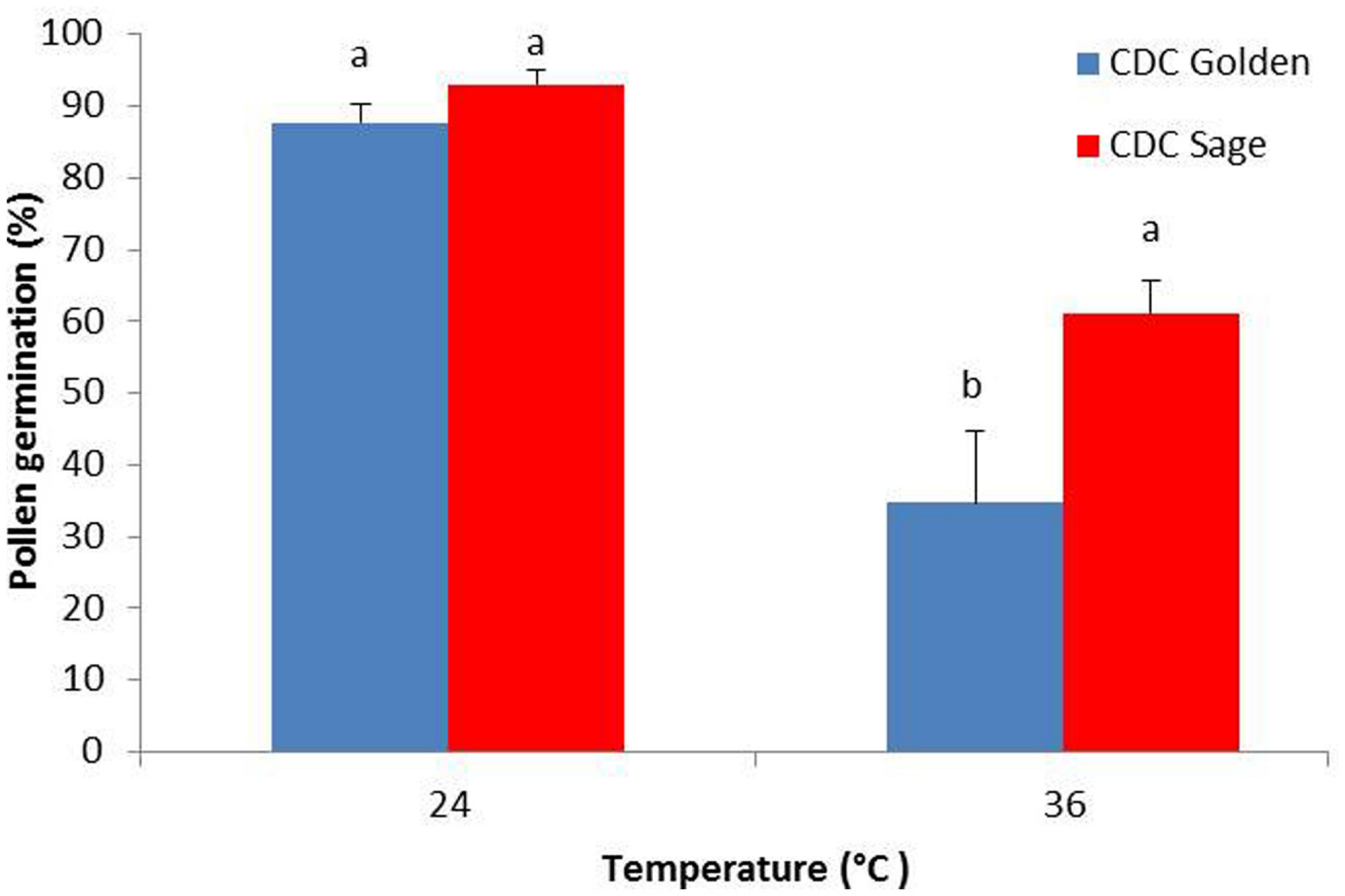
**Germination percentage of CDC Golden and CDC Sage pollen under control (24/18°C) and heat stress temperature (36/18°C).** Data are the mean of five replicates. At each temperature, treatments with different letters were significantly different (LSD test, *P* < 0.05).

The effect of heat stress on pollen germination was evaluated for both pea cultivars CDC Golden and CDC Sage (**Figure 1**). Results show that under normal conditions, there was no difference between both cultivars in the pollen germination percentage, whereas under heat stress conditions (36/18°C), percentage pollen germination was reduced by 30 and 55% in CDC Sage and CDC Golden, respectively. The heat stress reduced pollen germination in both cultivars and CDC Golden was affected more than CDC Sage.

### PEA POLLEN PROTEIN

**FIGURE 2:**
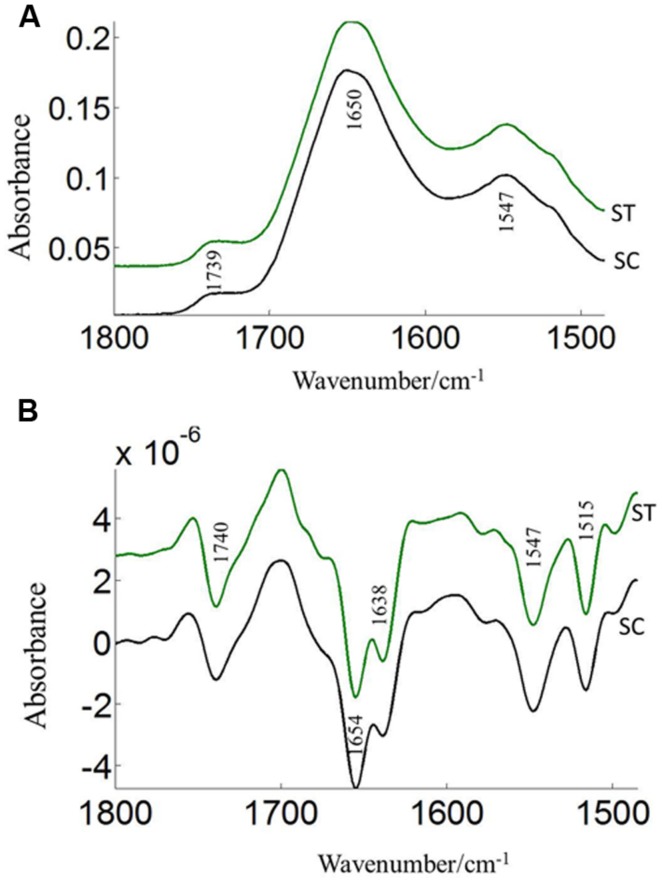
**Average protein (1800–1500 cm^–1^) absorbance **(A)** and respective second derivative **(B)** spectra of heat stressed (ST) and control (SC) pollen of CDC Sage.** Data are means of 15 individual pollen spectra per treatment combination.

**FIGURE 3:**
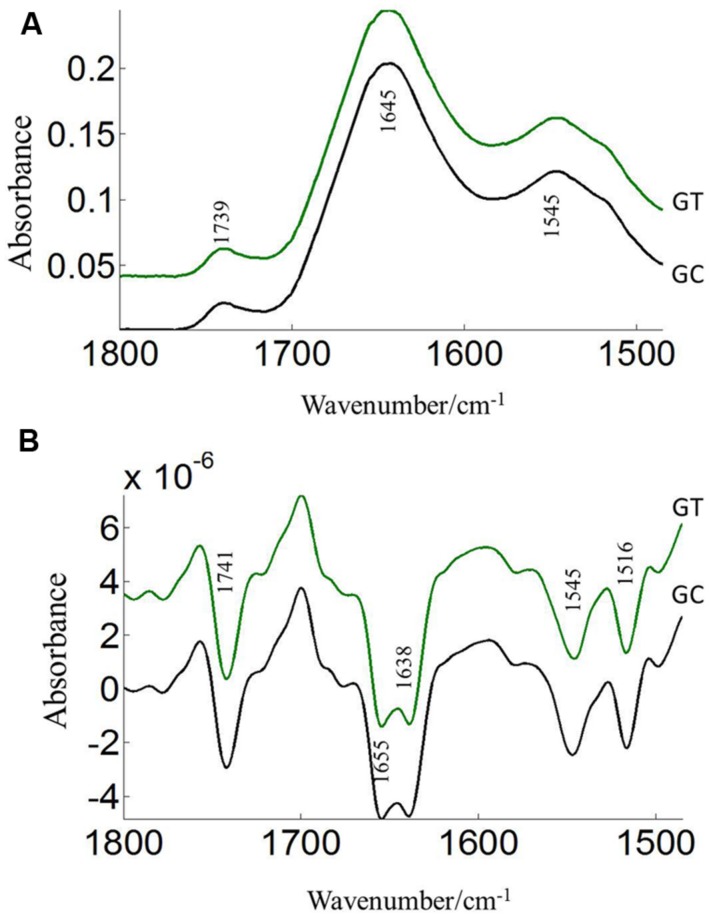
**Average protein (1800–1500 cm^–1^) absorbance **(A)** and second derivative spectra **(B)** of heat stressed (GT) and control (GC) pollen of CDC Golden.** Data are means of 15 individual pollen spectra per treatment combination.

The FTIR reveals molecular-vibrational transitions and provides characteristic information on molecular compositions (Griffiths, 1978). **Figures 2** and **3** show the absorbance (A) and second derivative spectra (B) of pollen from both cultivars, at control and high temperature. The amide I band (from 1700 to 1600 cm^-1^) is mainly associated with the C = O stretching vibration and is directly related to the protein backbone conformation (Sowa et al., 1991; Wolkers and Hoekstra, 1997; Fernandes et al., 2013). In the spectra of pollen, at least two main absorption peaks were observed at 1638 and 1654 cm^-1^ (**Figures 2B** and **3B**) in the amide I region. Both the peaks are sensitive to hydrogen bonding and coupling between transition dipoles of adjacent peptide bonds and hence, indicate different secondary structures (Sowa et al., 1991; Wolkers and Hoekstra, 1997). The absorption band positions identified in this study are in agreement with previous work on pollen (Wolkers and Hoekstra, 1995). In the literature (Wolkers and Hoekstra, 1995), the following bands in the amide I region are assigned: β-sheets/turn (1692–1690 cm^-1^), turn or loops (1674–1673 cm^-1^), helical segments and α-helices (1650–1654 cm^-1^), β-sheets (1638 cm^-1^) and protein side chains, as well as contribution of cell wall material at 1615 cm^-1^ (Wolkers and Hoekstra, 1995). Spectral differences between control pollen grains of the both cultivars were also observed.

Pollens from the two pea cultivars were subjected to two temperature regimes as described previously. The use of the extreme temperature of 36°C is justified by the semi-arid conditions in which pea cultivars are usually grown. To compare protein changes of control and heat stressed pea pollen grains, FTIR spectra were normalized using the C = O bond of the ester peak at 1740 cm^-1^. The intensity of the 1650–1654 cm^-1^ band was higher in control pollen and was reduced after heat stress for both cultivars (**Table 1**). In addition to lipids and protein secondary structures, **Table 1** show spectral differences in nucleotides (996 cm^-1^), carbohydrates (1053 cm^-1^), phosphate (*P* = 0, 1240 cm^-1^) between both CDC cultivars with/out heat stress.

**Table 1:** Integrated absorption peaks of O-H stretching-amide A (3315 cm^–1^), oxidative stress (= CH, 3015 cm^–1^), lipids (3000–2800 cm^–1^), amide I (1650 cm^–1^), phosphate (*P* = 0, 1240 cm^–1^), carbohydrates (1053 cm^–1^), nucleotides (996 cm^–1^), and other parameters such as esterification ratio and protein ratio in pollen of CDC Golden and CDC Sage under normal (24/18°C) and heat stress.

Peak position (cm^-1^)	Wavenumber range (cm^-1^)	Controls-pea pollen (24/18°C)		Heat stressed-pea pollen (36/18°C)	
		CDC Sage	CDC Golden	CDC Sage	CDC Golden
3315	3500–3200	115 ± 4**	82 ± 2	104 ± 4	87 ± 3
3015	3030–3000	2.7 ± 0.1	2.8 ± 0.1	2.8 ± 0.1	2.9 ± 0.1
3000–2800	3000–2800	3.0 ± 0.3	3.1 ± 0.2	3.1 ± 0.2	3.0 ± 0.2
1650	1700–1600	107 ± 4	112 ± 4	113 ± 4	110 ± 4
1240	1261–1200	0.83 ± 0.05	0.9 ± 0.1	1.1 3 ± 0.07	0.95 ± 0.06
1053	1090–1022	7.3 ± 0.3	7.0 ± 0.3	7.4 ± 0.2	7.0 ± 0.2
996	1010–960	2.6 ± 0.1	2.7 ± 0.1	2.5 ± 0.1	2.7 ± 0.1
1654/1638		1.18	0.76	0.95	0.71
1740 (1760–1720)		0.13	0.36	0.19	0.38
*ER		1.2	3.1	1.7	3.4

*Esterification ratio (%) = 1740/(1740 + 1650) × 100; **Standard errors (*n* = 15).

### PEA POLLEN PROTEIN SECONDARY STRUCTURES

**FIGURE 4:**
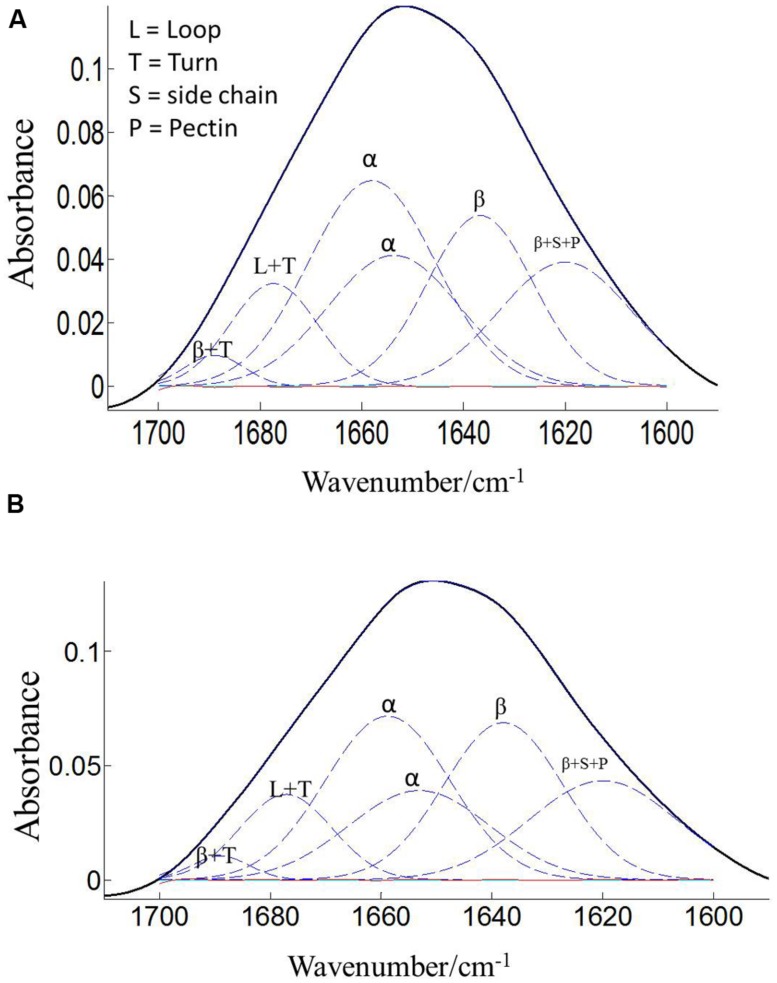
**Curve fitting of the amide I region (1700–1600 cm^–1^) of CDC Sage pollen at both treatment temperatures of 24/18°C **(A)** and 36/18°C **(B)**.** Data are means of 15 individual pollen spectra. L, T, S, P, α, and β indicate loop, turn, side chain, pectin, α-helical, and β-sheet, respectively.

**FIGURE 5:**
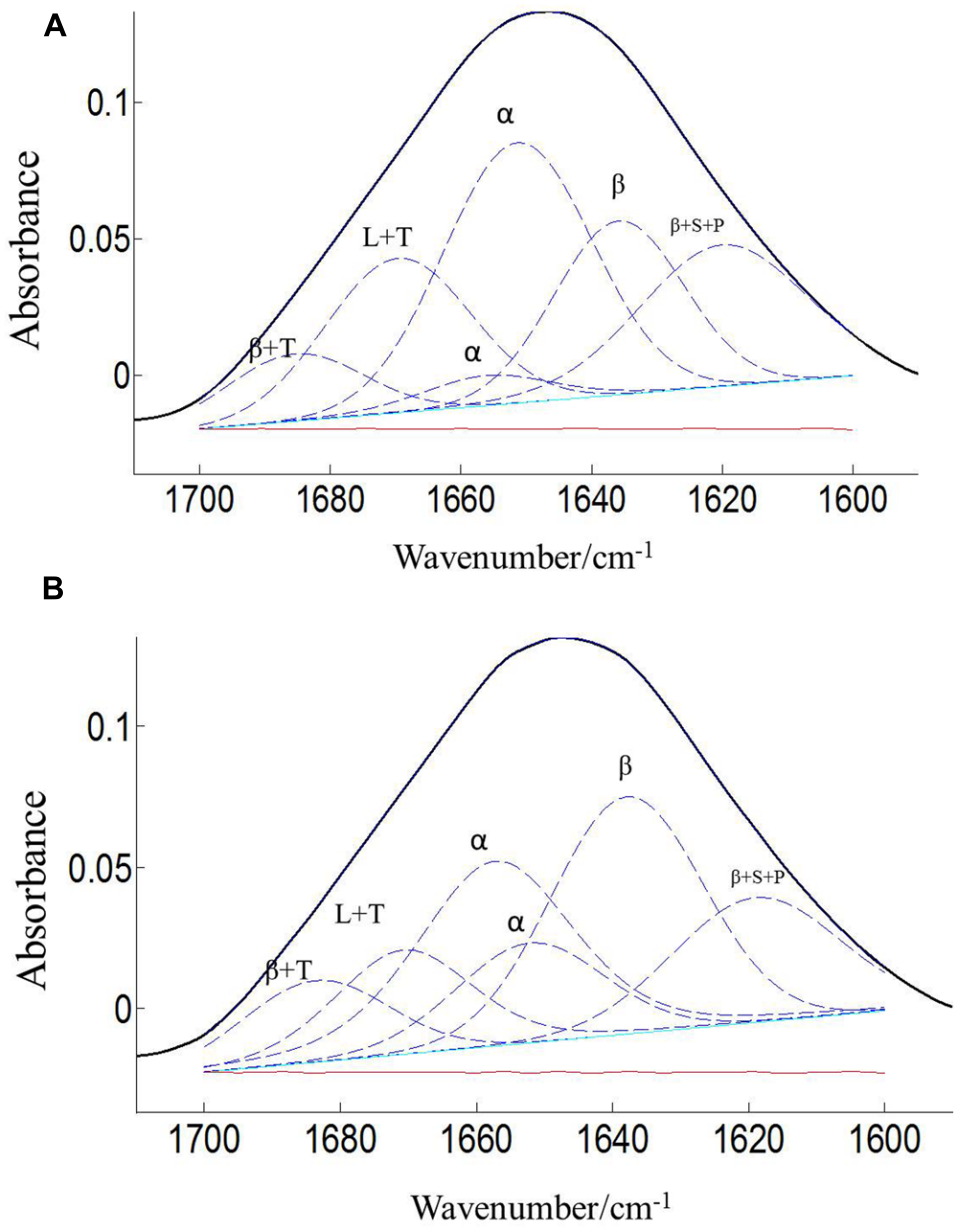
**Curve fitting of the amide I region (1700–1600 cm^–1^) of CDC Golden pollen at both treatment temperatures of 24/18°C **(A)** and 36/18°C **(B)**.** Data are means of 15 individual pollen spectra. L, T, S, P, α, and β indicate loop, turn, side chain, pectin, α-helical, and β-sheet, respectively.

The protein secondary structures of CDC Sage and CDC Golden pollen were also estimated for control and temperature treatment. **Figures 4** and **5** show the fitted amide I component bands of pollen protein in CDC Sage and CDC Golden. This curve fitting is based on the second derivatives of the original spectrum, which is a resolution enhancement technique to separate the overlapping bands. Before running the curve fitting we input the minimum and maximum wavenumber range for each peak based on the second derivative spectrum which is presented in the **Table 2**. The area of each peak was initialized manually based on the second derivative and than did the auto run by using the mixture of Lorentzian and Gaussian lineshapes which resulted the multi component peak and combination of this multi peak (blue) well overlapped with the original spectrum (**Figures 4** and **5**). The cpercent of each protein secondary structures is presented in **Table 2**.

**Table 2:** Selected area are used for curve fitting and protein secondary structure calculation on the basis of second derivative spectrum of pollen pea CDC Golden and CDC Sage.

Secondary structure content	Minimum value (cm^-1^)	Maximum value (cm^-1^)
β-sheets + turn	1682	1690
Loop + turn	1662	1678
α-helices	1648	1660
β-sheets	1630	1640
β-sheets + side chain + pectin	1610	1620

Bands were assigned to β-sheets, α-helical structure, and random coils or loops as above (**Figures 4** and **5**). The contributions by various protein secondary structures to the protein-rich matrix of pollen coat were estimated and interesting differences were observed in α-helical and β structures (**Table 3**). The contribution of α-helical structure was greater in CDC Sage than CDC Golden in both conditions (control and heat stressed) which may be one of the key components for sustaining heat tolerance. However, there was an increased reduction of α-helices in heat stressed CDC Sage. In contrast, the β-sheet structure was greater in CDC Golden pollen compared to CDC Sage under both conditions of control and high temperature. These differences were also reflected in the content of α to β ratio, which was much lower in CDC Golden compared to CDC Sage. Other secondary structural contributions such as random coils or loops in both cultivars were observed more in heat stressed pollen samples than in the control treatment. Heat treatment appeared to affect other protein secondary structures such as (turns and random coils) with a prominent increase in CDC Golden pollen (control and heat stressed), while no corresponding change was observed in CDC Sage pollen.

**Table 3:** Secondary protein structure data of pollen of CDC Golden and CDC Sage at control (24/18°C) and heat stress (36/18°C) conditions.

Protein structure	Control-Pea pollen (24/18°C)		Heat stressed-pea pollen (36/18°C)	
	CDC Sage	CDC Golden	CDC Sage	CDC Golden
β-sheet	41.3*	46.3	46.0	51.7
α-helix	48.6	35.3	43.6	36.2
others	10.1	18.4	10.4	12.0

Data were estimated from the average spectra of 15 individual pollen grains per treatment combination.

*Percentage with respect to 100% of total protein structures; others represent loops, turns, and pectin.

### PEA POLLEN LIPIDS

**FIGURE 6:**
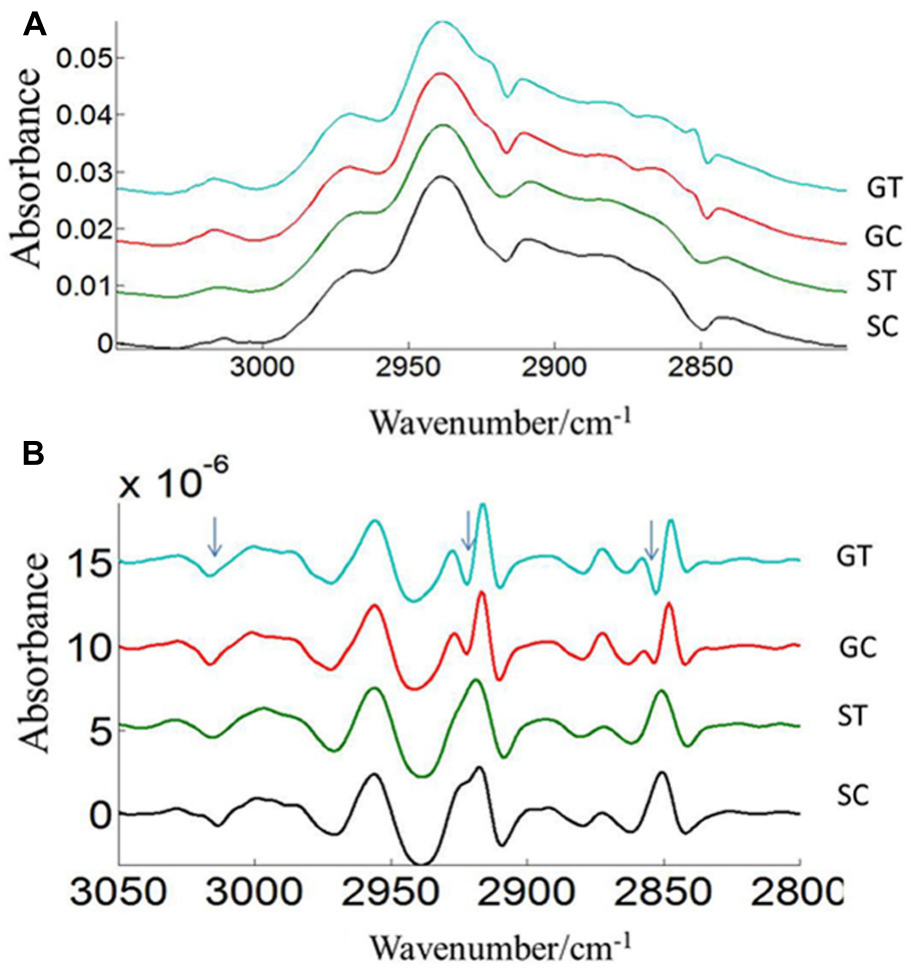
**Average lipid (3050–2800 cm^–1^) absorbance **(A)** and respective second derivative **(B)** spectra of heat stressed (GT, ST) and control (GC, SC) pollen of CDC Golden and CDC Sage.** Data are means of 15 individual pollen spectra. The arrow indicates changes in the lipid region due to heat stress.

**FIGURE 7:**
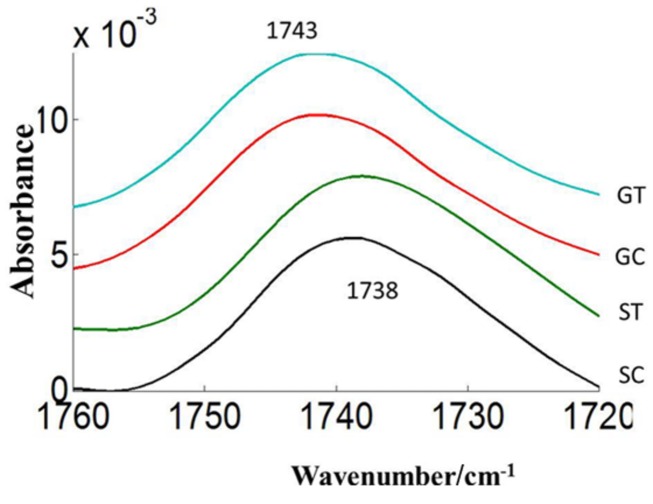
**Average carbonyl esters (1760–1720 cm^–1^) absorbance spectra of heat stressed (ST, GT) and control (SC, GC) pollen of pea cultivar CDC Sage and CDC Golden, respectively.** Data are means of 15 individual pollen spectra per treatment combination.

Three prominent areas in the lipid region were chosen for comparison between the two cultivars: 3050–3000 cm^-1^ (= CH), 3000–2800 cm^-1^ (CH_2_ and CH_3_ stretching vibrations of lipid acyl chain) and 1760–1720 cm^-1^ (carbonyl ester) to address the importance of lipids in CDC Sage and CDC Golden pollen (**Figures 6** and **7**) in control and heat stressed conditions. The band observed at 3015 cm^-1^ is assigned to = CH vibration. The bands observed at 2970 and 2876 cm^-1^ correspond to asymmetric and symmetric stretching vibration of CH_3_. Similarly, asymmetric and symmetric CH_2_ vibrations were observed at 2922 and 2852 cm^-1^. Results showed a strong difference in integrated absorbance of lipid regions between both pea cultivars and also due heat stress. The intensity of asymmetric and symmetric CH_2_ vibration of heat stressed CDC Golden pollen were considerably reduced in comparison to the control treatment (**Figure 6**). No such changes were observed in CDC Sage. Since the total lipid content of pollen from each pea cultivar was proportional to the IR absorbance (Griffiths, 1978), we compared the absorbance in the lipids region of both cultivars under control and heat stress conditions after normalization using the amide I region. In the control pollen, the lipid content was higher in CDC Golden than CDC Sage, but with heat stress, the lipid content increased by 0.067 [arbitrary units (a.u.)] in CDC Sage pollen and decreased by 0.096 a.u. in CDC Golden pollen. This suggests that lipids were more affected by heat stress in CDC Golden than CDC Sage (**Table 1**). Further, the band encoding oxidation stress located at ~3015 cm^-1^, was greater in CDC Golden pollen than in CDC Sage pollen (**Figure 6** and **Table 1**), even the CDC Golden may be more heat resistant via other mechanisms.

The carbonyl vibrations of esters occur at frequencies between 1760 and 1720 cm^-1^, and usually provide information about the polar interfacial regions of membrane lipids or pectin (Sowa et al., 1991). By inference, the ratio of areas of the bands at 1740 cm^-1^ over the sum of the areas of the bands at 1740 and 1650 cm^-1^ should be proportional to the degree of esterification. This method was applied to evaluate the degree of esterification of pectin cell walls from pollen of both pea cultivars CDC Golden and CDC Sage and to study the changes in the degree of esterification during heat stress. The peak of the ester carbonyl with the vibrational frequencies near to 1740 cm^-1^ of heat stressed pollen showed changes in intensity with heat stress on both pea cultivars (**Table 1**). This shows that a high degree of esterification in pollens of CDC Sage or CDC Golden under higher temperature stress with a slight difference between the two cultivars (**Figure 7**). A shift in the absorption bands in the carbonyl ester region was observed between CDC Golden and CDC Sage indicating changes in the lipids or pectin compositions between the two cultivars.

## DISCUSSION

### PEA POLLEN GERMINATION

Favorable pollen viability and seed set under heat stress are important traits for selection in breeding programs, and there is a strong correlation between pollen production and viability, dehiscence, and seed-set (Bita and Gerats, 2013). Temperature is one of the most important environmental stresses that may limit pollen growth and development (Hedhly et al., 2004; Rang et al., 2011). Temperature stress in the period preceding dehiscence results in more severe consequences for pollen viability than after dehiscence (Rang et al., 2011). Furthermore, both cold and heat stress during pollen development can negatively affect pollen viability depending on the species. The results from this study highlighted the impact of high temperature on pollen germination of two pea cultivars CDC Golden and CDC Sage. The germination rate of CDC Golden pollen decreased with increasing temperatures from 24 to 36°C. In CDC Sage, the pollen germination percentage was reduced with an increase in temperature from 24 to 36°C, although the effect was less than that observed in CDC Golden. This result indicated a difference in the response mechanisms of both pea cultivars facing heat stress, which may be related to pollen and pollen wall composition. The effect of heat stress during reproductive development has been thoroughly investigated in different crops. Temperatures between 20 and 25°C are reported to be optimal for pollen germination, pollen tube growth, and seed set (Sato et al., 2002; Jagadish et al., 2010; Rang et al., 2011). Surprisingly, raising the temperatures to 29°C and above drastically reduced the number of fruits formed and seeds set (Sato et al., 2002). Using male sterile plants, Peet et al. (1998) demonstrated that the effect was largely caused by defects in male reproductive organ development, while female organ development was less affected in tomato plants. In bean, when heat-sensitive and heat-tolerant genotypes were subjected to mild heat stress during development, pollen viability of the heat-sensitive genotype declined from 80% to below 10% after 10 days of heat treatment. The heat-tolerant genotype, however, still produced 60% viable pollen even after 24 days of stress. The effects of heat stress on pollen included alterations in the pollen wall composition and a reduction in the number of pollen grains that adhered to the stigma (Porch and Jahn, 2001).

### PEA POLLEN PROTEIN

The pollen coat is a layer of material made up of lipids and proteins which is present on the surface of the pollen wall and it would be sensitive to heat stress (Wolters-Arts et al., 1998). ATR–FTIR spectroscopy was used to compare the composition of the pollen coats of two pea cultivars CDC Sage and CDC Golden, and in response to heat stress. The FTIR spectra were used to assess the changes of protein. Comparison of the chemical structure of pollen for both pea cultivars, CDC Sage and CDC Golden revealed that they exhibit similar responses to heat stress, although the severity of the effect differed between the two pea cultivars. In general, the composition of amide I bands observed in whole pollen of both pea cultivars represented different types of protein secondary structures. The band at ~1654 cm^-1^ was assigned to α-helical structures, because α-helical vibrations are usually observed between 1650 and 1658 cm^-1^ (Haris and Chapman, 1992; Wolkers and Hoekstra, 1995). The curve fitting results of amide I region of pollen underline considerable differences between whole pollen grains of both cultivars in the presence and absence of heat stress. In control grains, the contribution of α-helical structures were more important in the whole pollen of CDC Sage (48.6%) than in CDC Golden (35.3%). This is in agreement with the contribution of α-helical structures in *in vitro* cattail pollen as reported previously by Wolkers and Hoekstra (1995). When whole pollen grains were subjected to *in vivo* heat stress, the contribution of α-helical structures was reduced from 48.6 to 43.6% in CDC Sage pollen but the content was still higher than that observed in CDC Golden. Wolkers and Hoekstra (1995) stated that the presence of α-helical domains protected bulk proteins in pollen against destructive effects of dehydration. The decrease of α-helical structures in the whole pollen of CDC Sage when subjected to heat stress may compensate for the reduced protein-water hydrogen band which may have explained its higher germination rate (60%). Therefore, the presence of low amounts of α-helical domains in whole pollen of CDC Golden may explain its sensitivity to heat stress as demonstrated by low pollen germination rate. In addition, the intensity of the oxidation band at ~3015 cm^-1^ was higher in CDC Golden than CDC Sage and increased with heat stress (**Table 1**), indicating that CDC Golden may have been affected by heat stress to a larger degree than CDC Sage. However, further analysis of cellular extracts of pollen from both pea cultivars is required and is planned in our future work to differentiate between membrane and cytoplasmic proteins as well as to test more varieties to quantify how changes in chemical molecular composition of pea cultivars pollens are linked to heat stress tolerance.

### PEA POLLEN LIPIDS

Lipids play an important role in pea pollen germination (Dickinson et al., 2000). The pea pollen surface lipid composition detected by ATR spectroscopy was likely from the pollen coat and sporopollenin on the exine layer. The exine lipid may have changed under heat stress in pea (cv. CDC Golden) and may have affected pollination events including pollen contact, adhesion and attachment of pollen to the stigma. Such a lipid change can affect pollen hydration, pollen germination, pollen tube growth through the stigma, style, and ovary, and fertilization of the ovule. This study demonstrated a difference in the lipids region due to heat stress and treated pollen from CDC Golden which was not observed in CDC Sage (**Figure 6**). This suggests that lipids were more affected by heat stress in CDC Golden than CDC Sage. This was confirmed by the considerable reduction of asymmetric and symmetric CH_2_ peaks in heat stressed samples (**Figure 6B**). Porch and Jahn (2001) have remarked that high temperatures may result in the structural modification of the exine layer of common bean pollen. The external pollen cell wall, exine, protects the pollen grain from environmental damage and dehydration and also facilitates pollen-stigma interactions, pollen hydration, and release of the pollen tube to effect fertilization (Mach, 2012). The exine is largely formed from acyl lipids and phenylpropanoid precursors. These two main components that are synthesized in the tapetum form the stable biopolymer sporopollenin structure of the exine (Piffanelli et al., 1998). Both the exine coating of the pollen grain and the dry surface of the stigma affect pollen adhesion to the stigma (Herscovitch and Martin, 1989). After pollen adhesion to the stigma and formation of a hydration lens, the outer exine coating flows out and forms a “foot” to establish attachment at the point of pollen-stigma contact (Scott, 1994). Availability of high amounts of lipids are mandatory for pea pollination, including different steps from pollen germination to pollen-attachment and adhesion stigma contact. Consequently, the difference in lipid amounts in whole pollen between the two pea cultivars after heat stress may contribute to a better explanation of pollen germination percentage differences observed in this study. This observation is in agreement with a greater amount of amide I in pollen of CDC Sage compared to CDC Golden that may prevent the loss of hydrogen bonds during heat stress which is highly correlated with the results shown for α-helical domain structures. In a recent study, Zimmermann and Kohler (2014) determined that IR spectra showed larger inter-annual variations in pollen composition of pine trees and these variations were predominantly due to differences in lipid content. They determined that pollen lipid patterns had a strong correlation with temperature profiles prior to pollination starting dates. Similarly, Wolters-Arts et al. (1998) reported that lipids are an essential compound needed for pollen tubes to penetrate the stigma, and in the presence of these lipids pollen tubes can even penetrate leaf cuticles. The authors further proposed that lipids direct pollen-tube growth by controlling the flow of water to pollen in species with dry and wet stigma. Accordingly, it would be interesting to further investigate the role of components of the exudate or pollen coat of pea cultivars for pollen tube penetration under heat stress. On the other hand, the spectral absorbance of ester carbonyl regions that are mainly known as pectin contributors were only slightly changed in pollen with increasing temperature in both pea cultivars, suggesting that pectin metabolism did not play a central role in pollen tolerance to heat stress, but its contribution was likely prominent in subsequent pollen tube growth and viability as previously shown by Tian et al. (2006).

## CONCLUSION

Pea has heat resistant mechanisms associated with pollen grains. Genotypes with pollen that has heat resistance have maintained pollen vigor and increased pollen germination, allowing for a greater success in fertilization. Two such mechanisms were evident in the physical structure of pollen, by maintained protein and lipid integrity of the coat. The ATR–FTIR spectroscopy was well adopted as a non-destructive chemical tool for investigating biochemical differences between the pollen grains of two pea cultivars and under environmental stress conditions *in vitro* like heat stress. Pollen from cultivar CDC Sage had more α-helical structures of protein than CDC Golden pollen, which had more β-sheets. The genotypic differences in pollen between compositions were detected in the lipid regions, and a prominent change was revealed in asymmetric and symmetric CH_2_ bands in CDC Golden pollen under heat stress. The bands for lipid, protein, and carbohydrates were different between the two cultivars and varied with heat stress. Additionally, the band corresponding to oxidative stress was much higher in CDC Golden pollen than CDC Sage pollen with heat stress. These findings may explain the difference in pollen germination between both pea cultivars under heat stress. Our results underline changes in the composition of major biomolecules (proteins, lipids, and carbohydrates) of pea pollen grains, which may be indicators of heat stress tolerance, and results may also indicate dual or separate roles of protein and lipids. Therefore, further investigations are planned to confirm the correlation between the *in vivo* pollen viability and pollen molecular composition by assessing the viability of pollen after *in vivo* heat stress on more pea varieties.

## AUTHOR CONTRIBUTIONS

Rachid Lahlali, Yunfei Jiang, Chithra Karunakaran, and Rosalind Bueckert conceived this research. Rachid Lahlali, Chithra Karunakaran, Yunfei Jiang, and Saroj Kumar collected and analyzed output data. Xia Liu and Ferenc Borondics helped in FTIR setup and data collection. Emil Hallin and Rosalind Bueckert supervised the work. Rachid Lahlali and Saroj Kumar wrote the manuscript and all authors contributed to the manuscript revision.

## Conflict of Interest Statement

The authors declare that the research was conducted in the absence of any commercial or financial relationships that could be construed as a potential conflict of interest.
